# Acceptability and validity of HPV self‐sampling for cervical cancer screening among women living in different ecological settings in India

**DOI:** 10.1002/ijc.35222

**Published:** 2024-10-16

**Authors:** Gauravi A. Mishra, Sharmila A. Pimple, Kavita V. Anand, Vasundhara Y. Kulkarni, Anil S. Patil, Sanjay K. Biswas

**Affiliations:** ^1^ Department of Preventive Oncology Centre for Cancer Epidemiology (CCE), Tata Memorial Centre, Homi Bhabha National Institute (HBNI) Mumbai India; ^2^ Department of Microbiology Tata Memorial Hospital, Tata Memorial Centre, Homi Bhabha National Institute (HBNI) Mumbai India

**Keywords:** cervical intraepithelial neoplasia, cervix cancer, community health education, early detection of cancer, human papillomavirus

## Abstract

India records one fifth of global cervical cancer burden. Unlike human papillomavirus (HPV) self‐sampling, other screening methods may cause discomfort and embarrassment. This study aimed to investigate attitudes, acceptability, barriers, predictors, effective modality of instructions, and validity of HPV self‐sampling among Indian women residing in varied settings and different literacy levels. This is community‐based interventional study among Indian women 30–55 years, residing in urban slums (500), urban non‐slums (500), and rural (600) settings with varied washroom facilities and privacy, to collect self‐samples. Each group was subdivided into two arms; in one women received education with pamphlets and other with health education program (HEP). Study involved enlisting eligibles, obtaining informed consents and conducting personal interviews to collect baseline data. Self‐samplers were distributed with instructions (pictorial pamphlets in one group and HEP in other) regarding usage, storage and return. Willingness to use self‐samplers, refusals, experiences, and so forth were captured. Post‐intervention knowledge, attitudes, practices was recorded. HPV reports were distributed. Women with positive result on either test were offered further management. Acceptance rate of self‐sampling was 99.2%, 97%, and 98.8% and HPV positivity was 7%, 7.8%, and 8.5%, respectively among urban non‐slum, urban slum and rural women. Agreement rate between health personnel collected and self‐collected samples was 96.5% in pamphlet and 93.2% in HEP arm. Major barriers of self‐sampling were lack of confidence about performing self‐test correctly, fear that test would be painful and anxiety about test results. HPV self‐sampling has good acceptability among Indian women and good agreement with health personnel collected samples.

## Graphical abstract

What's new?

About 20% of cervical cancer cases worldwide occur in India, which is one of the world's most religiously and socioeconomically diverse countries. Against this backdrop of diversity, the extent to which women accept human papillomavirus (HPV) self‐sampling for cervical screening is unknown. Here, HPV self‐sampling was investigated among Indian women in different living conditions. Data show that in urban non‐slums, urban slums, and rural settings, self‐sampling was accepted by 99.2%, 97.0%, and 98.8% of women, respectively. Education about self‐collection was associated with similar acceptance across groups, suggesting that heightened awareness and access to information facilitate HPV self‐sampling.

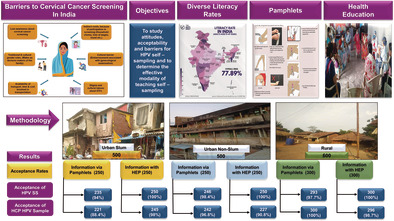

## 1 INTRODUCTION

Cervical cancer is the fourth most common cancer among women globally. Over 662,301 cases and 348,874 deaths due to cervical cancer occurred in 2020. Around 60% cervical cancer cases and deaths are from Asia and about one fifth of the global burden is from India.^1^ Various screening tools are available for early detection of cervical cancer. Women in developed countries undergo regular screening due to which women are detected in cervical precancerous stage. Appropriate management of precancers leads to virtual cure. Hence, these countries have low incidence and mortality due to cervical cancer. Though India has the highest number of cervical cancer cases, <3% of Indian women between 30 and 49 years are ever screened, (2.2% in urban and 1.7% in rural).^2^ Various factors including poor health awareness, competing priorities, limited accessibility to health care facilities, reluctance in acceptance of screening procedures, preference to be screened by female health personnel, lack of decision‐making capacity among women, and so forth contribute to poor screening rates. There is a need to introduce solutions that would circumvent these issues.

Persistent infection with carcinogenic human papilloma virus (HPV) is responsible for virtually all cases of cervical intraepithelial neoplasia (CIN) and invasive cancers.^3^ The commonly used screening methods to detect CIN and cancers are cytology, HPV DNA test, and visual inspection with acetic acid (VIA). These screening tests are performed by health care personnel (HCP) and require per speculum insertion. Cytology‐based screening is labor‐ and time‐intensive and lacks sensitivity and reproducibility to detect CIN or cervical cancer.^4^, ^5^ VIA has been investigated in two randomized controlled trials from India and has shown to reduce mortality due to cervical cancers.^6^, ^7^ It has been incorporated in the National Cancer Control Programmes in several countries in Asia and Africa.^8^ The operational guidelines of the National Programme for Prevention and Control of Non‐Communicable Diseases (NP‐NCD) in India also recommend VIA‐based cervical cancer screening.^9^ World Health Organization (WHO) recommends use of HPV test for primary cervical cancer screening.^10^ Most developed countries have transitioned or are transitioning from cytology to HPV test for primary screening of cervical cancers. Europe, The Netherlands, and Turkey have fully transitioned from cytology to HPV‐based screening while Italy, Sweden, and Finland have implemented HPV screening in several regions, and several other European countries are at various stages of implementation.^11^ Recent American Cancer Society's ACS 2020 guidelines also preferentially recommends HPV test.^12^

HPV‐based screening tests are reproducible, have higher sensitivity for detecting high‐grade CIN, and have very good negative predictive values (NPVs) as compared to cytology.^13^, ^14^ Hence screening intervals can be lengthened, thus requiring fewer screening rounds. Current standard method of HPV DNA screening necessitates women to visit health care facility which may not be accessible, thus making self‐sampling possible alternative. In addition, major concerns of women with clinic‐based approach are that, women may perceive procedures as unpleasant, embarrassing, preference to be screened by female health care provider, physical, and/or psychological discomfort associated with the test and time off required from work in order to attend screening clinic.^15^

Several studies in different countries have demonstrated self‐sampling for HPV to be a cost effective, easy, feasible, and well‐accepted method with high accuracy.^16^, ^17^ Self‐sampling has the advantage of not requiring a vaginal speculum examination, thus reducing the discomfort that may make screening unattractive, even among women who have access to health care. Also, women can perform the test in privacy of their home at convenient time. There, however, may be several unique acceptability issues considering wide range of Indian settings. Low literacy levels among women in certain areas may influence the uptake and precision in collecting the sample for HPV‐self sampling. Modalities of communication and instructions to collect self‐samples may influence the accuracy of results.

This study aims to investigate the knowledge, attitudes, practices (KAP), feasibility, acceptability, and accuracy of HPV self‐sampling for cervical cancer screening in resource‐constraint country like India, in an attempt to increase the coverage of cervical cancer screening. The study also aims to enhance our understanding toward determinants of acceptability and barriers of self‐collection for HPV DNA testing in different settings in India with three populations groups (urban non‐slums, urban slums, and rural), wherein half of the participant women are provided information regarding cervical cancer and method of self‐collection using pamphlets alone and other group comprising rest half of women are offered this information through a health education program (HEP). The other component of the study was to investigate the agreement rates between the self‐collected and the HCP‐collected samples in these various subgroups. The trial was approved by the Institutional Ethics Committee and registered with the clinical trials registry.

## 2 MATERIALS AND METHODS

**FIGURE 1:**
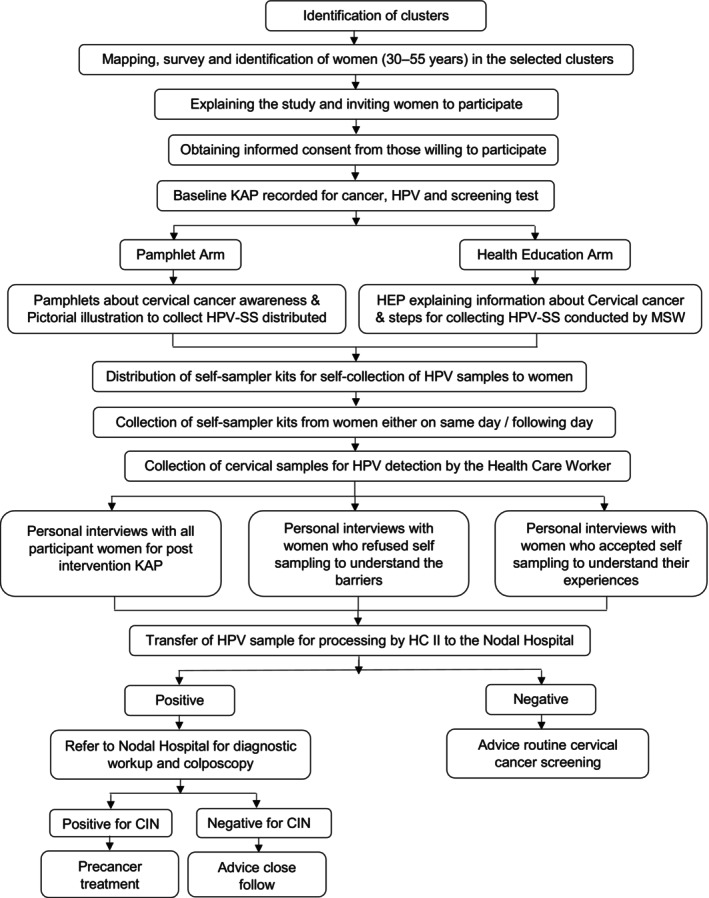
Flowchart of study methodology. CIN, cervical intraepithelial neoplasia; HPV, human papillomavirus; KAP, knowledge, attitudes, practices; MSW, medical social worker.

This is a community‐based interventional study among 1600 sexually active women in age group 30–55 years, residing in three different settings (with different housing conditions, washroom facilities, and privacy) in Maharashtra, India; (a) Urban slum area – Mumbai City (500 women), (b) Urban Non‐slum area – Mumbai City (500 women), and (c) Rural area – Thane District (600 women). Pregnant women, women with frank cervical cancer, and women with hysterectomy were excluded from the study. The trial was initiated in April 2021 and was planned for 18 months duration. The study flow chart is represented in Figure 1. Initially, staff comprising mainly of medical social workers (MSWs) and health care workers (HCWs) were recruited and trained. Main role of MSWs was conducting baseline surveys, delivering HEP/distribution of pamphlets, conducting personal interviews, recording data on the proforma and the main role of HCWs was collecting HPV samples and transporting to the nodal hospital.

Three communities were selected and meetings were held with local leaders to enlist their support. The eligible women were contacted by door‐to‐door visit and the project was explained. Informed consent was obtained from those who were willing to participate and they were enrolled. Personal interviews were conducted to collect the baseline information about KAP regarding cervical cancer, screening and HPV. This information was recorded on a structured questionnaire.

Women from each of the three clusters were divided into two arms (viz., HEP arm and pamphlet arm) again by cluster randomization. Women in the HEP arm were invited to specially organized camps in their neighborhood for HEP, explaining them the risk factors, methods of early detection, prevention, signs and symptoms of cervical cancer, method of use (steps demonstrating the technique to collect HPV‐SS), storage and return of used self‐samplers. HEP was conducted in local language using flip charts/posters by MSWs. Primary awareness was created for women in pamphlet arm using art‐based pamphlets with self‐explanatory pictorial illustrations, demonstrating the steps involved in collecting HPV‐SS. These pamphlets form resources developed under the study. These were distributed by MSW to enrolled women in pamphlet arm in each cluster. Also, another information pamphlet in local language was distributed explaining risk factors, methods of early detection, prevention, signs and symptoms of cervical cancer and storage and return of used self‐samplers. HPV self‐samplers were distributed to participant women in all clusters and MSWs instructed women to collect the self‐samples, store it at room temperature and return it to the clinic; either on the same day or following day as per the convenience of the women. When the women came to return the samplers, their willingness to use self‐sampler or the reasons for their refusal were captured using a structured questionnaire for all groups of women. Any specific comments, barriers, and motivators for self‐sampling were recorded. During this visit, HCW collected HPV samples for the women. It was also collected for women who refused self‐sampling but were ready for HCW‐collected sample. The cervico‐vaginal samples for both, self and HCW sampling were collected using DigeneHC2 NA Collection Device and then placed in 1 mL of specimen transport medium containing PreservCyt solution. Information about the post‐intervention KAP regarding cervical cancer, screening, and HPV was again recorded on a structured questionnaire for all women.

The self‐sampler kits after collection were sent to the nodal hospital for analysis by Hybrid capture System II {Digene}, on a Qiagen platform, which detects 13 different high‐risk HPV types {16, 18, 31, 33, 35, 39, 45, 51, 52, 56, 58, 59, 68}.^18^ The reports obtained as positive or negative for HPV, were distributed to the women after about 2 weeks of collection. The women who tested positive on either test were contacted and invited to the nodal hospital where they were offered further investigations, Colposcopy, and necessary work‐up as per departmental service protocol. The women detected with precancers and cancers received treatment free of cost with the project funds at the nodal hospital.

Sample size: Assuming HPV prevalence among the women to be 7%–10% based on the published reports,^19^, ^20^ and concordance rate of 80%, at 95% confidence interval (CI) and power of 80%, between samples collected by HCW and self‐collected samples, around 1600 women were needed to undergo HPV testing by both the methods.

The data were entered in IBM SPSS Statistics for Windows, Version 25.0 (IBM Corp., Armonk, NY) software. Checks for consistency, data safety, data errors, and analysis were carried out at regular intervals. Both descriptive and inferential statistics were generated for describing variables under the study objectives. Categorical variables were summarized using frequencies and percentages. The normality of continuous variables was assessed through visual inspection of P–P and Q–Q plots, as well as histograms. Comparisons between demographic variables and study arms were conducted using Student's *t*‐test for continuous variables and Chi‐square test or Fisher's exact test was used for categorical values. The chance corrected agreement of the self‐ and health worker‐collected samples was assessed by Cohen's kappa statistic along with a 95% CI Cohen's kappa (*k*) value near zero represented agreement being due to random chance, 0.01–0.20 represented slight agreement; 0.21–0.40, fair agreement; 0.41–0.60, moderate agreement; 0.61–0.80, substantial agreement; and 0.81–1.00 almost perfect agreement.^21^ The comparison of pre‐ and post‐intervention results of KAP was assessed by using Mc‐Nemar test. The prevalence of HPV was calculated, along with its 95% CI using the Clopper‐Pearson method. The diagnostic test characteristics of HPV‐SS and HPV testing by HCP for CIN grades 1 and 2 were evaluated using sensitivity, specificity, false positive rate (FPR), false negative rate (FNR), positive predictive value (PPV), and NPV. All tests were carried out using an α = .05 level of significance.

## 3 RESULTS

Eligible women were identified in each cluster, 807 in urban slum cluster, 1859 in urban non‐slum cluster, and 1276 in rural cluster. Amongst these, 500, 500, and 600 women from each of these clusters respectively accepted the invitation and were enrolled. The baseline characteristics of participants in the three settings of the study are depicted in Table 1 and details also explained in Table S1. The median age of women enrolled in the trial was 40 years. The median age of marriage was 20 years. Fewer women from urban non‐slum cluster were illiterate (2.6%) as compared to rural women (13.50%) and women from urban slums (11.40%). Though majority women belonged to Hindu religion (overall 85.75%), there were high proportion of Buddhist women (34.40%) in urban slums compared to other clusters. Around 75% of women in each cluster were homemakers, while high proportion (18.83%) of women in rural setting were manual laborers. Overall family income was below 10,000 in 50% women; however, in rural area nearly 69% women had family income less than 10,000. Family history of cancer was present in 18.20% non‐slum women as opposed to only 9.33% rural women. Overall 97.13% women were ever pregnant with median age at first child delivery 22 years and history of two live births. Median age of menarche was 14 yrs. and 75.75% women were premenopausal. The use of contraception was reported to be 51.13% and 13.63% women had consulted medical center for some gynecological issue in past.

**TABLE 1:** Distribution of sociodemographic and risk factor variables among enrolled women in different settings.

Variables	Overall *N* (%) 1600	Urban non‐slum *n* (%) 500	Urban slum *n* (%) 500	Rural *n* (%) 600
Sociodemographic factors				
Age (years)				
30–35	482 (30.13)	119 (23.80)	181 (36.20)	182 (30.33)
36–40	414 (25.87)	128 (25.60)	137 (27.40)	149 (24.83)
41–45	319 (19.94)	117 (23.40)	87 (17.40)	115 (19.17)
46–50	250 (15.63)	82 (16.40)	67 (13.40)	101 (16.83)
51–55	135 (8.43)	54 (10.80)	28 (5.60)	53 (8.84)
Mean age (SD)	40.14 (6.77)	41.19 (6.77)	39.04 (6.52)	40.18 (6.84)
Median age (IQR)	40 (35–45)	41 (36–46)	38 (34–44)	40 (35–46)
Mean age at marriage (SD)	20.92 (4.47)	22.95 (4.76)	19.94 (4.13)	20.04 (3.90)
Median age at marriage (IQR)	20 (18–24)	22 (20–26)	19 (18–22)	20 (18–22)
Education				
Illiterate/literate without formal education	151 (9.44)	13 (2.60)	57 (11.40)	81 (13.50)
Primary (1–4)	94 (5.88)	17 (3.40)	38 (7.60)	39 (6.50)
Secondary (5–10)	970 (60.62)	260 (52.00)	305 (61.00)	405 (67.50)
Higher secondary (11–12)	186 (11.63)	82 (16.40)	49 (9.80)	55 (9.17)
Sr. College (13–15) undergraduates	94 (5.87)	60 (12.00)	28 (5.60)	6 (1.00)
Graduates and above	105 (6.56)	68 (13.60)	23 (4.60)	14 (2.33)
Religion by birth				
Hindu	1372 (85.75)	495 (99.00)	304 (60.80)	573 (95.50)
Muslim	32 (2.00)	0 (0.00)	16 (3.20)	16 (2.67)
Buddhist	180 (11.25)	2 (0.40)	172 (34.40)	6 (1.00)
Christian/Jain/Sikh	16 (1.00)	3 (0.60)	8 (1.60)	5 (0.83)
Occupation				
House wife	1215 (75.94)	375 (75.00)	391 (78.20)	449 (74.83)
Manual labor	197 (12.31)	35 (7.00)	49 (9.80)	113 (18.83)
Service	153 (9.56)	68 (13.60)	57 (11.40)	28 (4.67)
Self‐employed	35 (2.19)	22 (4.40)	3 (0.60)	10 (1.67)
Monthly family income (in Rs.)				
≤10,000	799 (49.94)	189 (37.80)	197 (39.40)	413 (68.83)
>10,000	801 (50.06)	311 (62.20)	303 (60.60)	187 (31.17)
Risk factors				
Family history of cancer				
Yes	201 (12.56)	91 (18.20)	54 (10.80)	56 (9.33)
No	1399 (87.44)	409 (81.80)	446 (89.20)	544 (90.67)
Pregnancy history				
Yes	1554 (97.13)	479 (95.80)	486 (97.20)	589 (98.17)
No	46 (2.87)	21 (4.20)	14 (2.80)	11 (1.83)
Mean age at first delivery (years) (SD)	23.06 (4.18)	24.78 (4.33)	21.93 (4.08)	22.58 (3.68)
Median age at first delivery (years) (IQR)	22 (20–26)	24 (21.25–27.75)	21 (19–24)	22 (20–25)
Median number of live births (IQR)	2 (2–3)	2 (1–2)	2 (2–3)	2 (2–3)
Menstrual status				
Premenopausal	1212 (75.75)	386 (77.20)	388 (77.60)	438 (73.00)
Perimenopausal	133 (8.31)	41 (8.20)	45 (9.00)	47 (7.83)
Postmenopausal	255 (15.94)	73 (14.60)	67 (13.40)	115 (19.17)
Median age at menarche (IQR)	14 (13–14)	14 (13–15)	14 (13–14)	14 (13–14)
Median age at menopause (IQR)	45 (41–47)	46 (44–49)	45 (41–47)	44 (40–46)
Undergone check‐up for any gynecological problems				
Yes	218 (13.63)	90 (18.00)	84 (16.80)	44 (7.33)
No	1382 (86.37)	410 (82.00)	416 (83.20)	556 (92.67)
History of contraceptive use				
Yes	818 (51.13)	260 (52.00)	267 (53.40)	291 (48.50)
No	782 (48.87)	240 (48.00)	233 (46.60)	309 (51.50)

**FIGURE 2:**
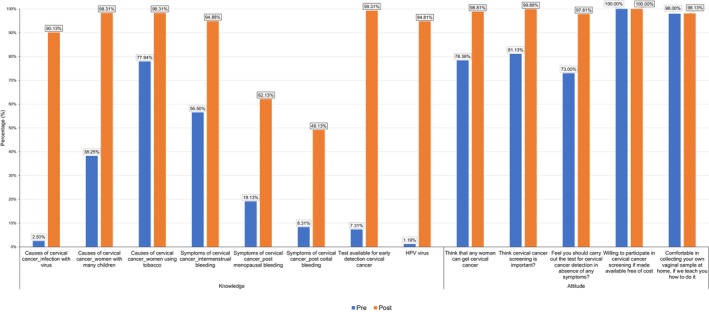
Overall comparison of pre‐intervention and post‐intervention knowledge and attitudes toward cervical cancer and screening. HPV, human papillomavirus.

Figure 2 depicts the overall comparison of pre‐intervention KAP with the post‐intervention KAP among women enrolled in the trial. In pre‐intervention period, overall, 78.38% women reported perceived risk of cervical cancer, 73% were aware of the importance of screening, but only 5.13% women had undergone cervical cancer screening in the past. Among the screened women, screening test was advised mainly by family physician (59.76%), followed by community health worker (37.80%). Rest 2.44% women underwent screening due to awareness created on social media or advice by friends. There was a significant increase (*p* < 0.001) in knowledge pertaining to HPV and cervical cancer after intervention across all three settings irrespective of the modalities of generating awareness (pamphlet vs. HEP by MSWs). There was also a significant positive change in attitude (*p* < .001) for screening across all three settings (Table S2).

Overall 1531 (95.69%) women accepted collection of samples by HCP, while 69 women refused. Most rural women accepted HCP examination and collection of HPV self‐samples (100% participation in rural pamphlet arm and 98.67% in HEP arm—4 refusals). Among urban women, participation was least in slum setting in pamphlet arm (88.40%), followed by non‐slum HEP arm (90.80%). While participation of women for HCP samples in urban non‐slum pamphlet arm (96.80%) and urban slum HEP arm (98%) was fairly good.

Totally, 1574 (98.38%) women collected self‐samples. More women accepted self‐sampling (1574) as compared to HCP‐collected samples (1531). The overall acceptance rates of self‐sampling were 96.75% in the pamphlet arm; with acceptance of 98.40% in urban non‐slum, 94% in urban slum and 97.67% in rural settings. All 100% women in HEP arm; in all three settings accepted self‐sampling. Thus, all 26 women who denied self‐sampling belonged to the pamphlet arm (3.25% refusals). Fifteen women from urban slum setting, four from urban non‐slum setting and seven from rural setting refused self‐sampling. The distribution of self‐sampling acceptance by literacy level across settings is demonstrated in Table S3.

Major barriers for self‐sampling reported amongst these women were lack of confidence about performing the self‐test correctly, fear that test would be painful and/ or could cause injury, unable to understand steps in pictorial illustrations (mainly among rural women), anxiety about test results, absence of privacy at home (mainly among urban slum women) and lack of perceived risk of cervical cancer.

Totally, both HCP collected samples and self‐collected samples were obtained for 1505 (94.06%) women. The agreement between HCP‐collected samples and self‐collected samples is depicted in Table 2. There was moderate agreement between the HPV‐HCP collected samples and HPV‐SS; 94.82% (*k* = 0.508 [95% CI = 0.458–0.559, *p* = 1.5124E‐86]). Agreement was 96.47% (*k* = 0.588 [95% CI = 0.516–0.660, *p* = 1.59992E‐57]) in pamphlet arm and 93.23% (*k* = 0.454 [95% CI = 0.383–0.525, *p* = 2.32049E‐36]) in HEP arm. Overall, fairly good agreement between the self‐collected samples and HCP collected samples was noted in all clusters in both HEP and pamphlet arms.

**TABLE 2:** Participation rates and agreement between HCP‐collected samples and self‐collected samples for HPV testing in various settings and with different modalities of communication regarding method for collecting self‐samples.

Site	Arm (*N*)	Participated in HCP‐collected sample *N* (%)	Participated in SS *N* (%)	Self‐collected sample	HCP‐collected sample			Agreement rate	HPV positivity agreement	Kappa statistics	95% CI	*p*‐Value	Conclusion
					Negative	Positive	Total	(%)	(%)				
Urban non‐slum	Pamphlet (250)	242 (96.80)	246 (98.40)	Negative	228	2	230	97.48	40.00	0.559	0.433–0.684	2.92775E‐18	Moderate agreement
				Positive	4	4	8						
				Total	232	6	238						
	HEP (250)	227 (90.80)	250 (100)	Negative	203	10	213	91.63	20.83	0.300	0.170–0.430	6.0191E‐06	Fair agreement
				Positive	9	5	14						
				Total	212	15	227						
	Total (500)	469 (93.80)	496 (99.20)	Negative	431	12	443	94.62	26.47	0.390	0.300–0.481	3.7073E‐17	Fair agreement
				Positive	13	9	22						
				Total	444	21	465						
Urban slum	Pamphlet (250)	221 (88.40)	235 (94)	Negative	190	5	195	96.12	50.00	0.646	0.510–0.782	1.26384E‐20	Substantial agreement
				Positive	3	8	11						
				Total	193	13	206						
	HEP (250)	245 (98)	250 (100)	Negative	223	6	229	94.69	40.91	0.552	0.427–0.678	5.11214E‐18	Moderate agreement
				Positive	7	9	16						
				Total	230	15	245						
	Total (500)	466 (93.20)	485 (97)	Negative	413	11	424	95.34	44.74	0.593	0.501–0.686	2.002E‐36	Moderate agreement
				Positive	10	17	27						
				Total	423	28	451						
Rural	Pamphlet (300)	300 (100)	293 (97.67)	Negative	273	4	277	95.90	40.00	0.550	0.437–0.664	1.5931E‐21	Moderate agreement
				Positive	8	8	16						
				Total	281	12	293						
	HEP (300)	296 (98.67)	300 (100)	Negative	265	12	277	93.24	35.48	0.488	0.375–0.601	3.2608E‐17	Moderate agreement
				Positive	8	11	19						
				Total	273	23	296						
	Total (600)	596 (99.33)	593 (98.83)	Negative	538	16	554	94.57	37.25	0.514	0.433–0.595	1.0368E‐35	Moderate agreement
				Positive	16	19	35						
				Total	554	35	589						
Total	Pamphlet (800)	763 (95.38)	774 (96.75)	Negative	691	11	702	96.47	43.48	0.588	0.516–0.660	1.5999E‐57	Moderate agreement
				Positive	15	20	35						
				Total	706	31	737						
	HEP (800)	768 (96)	800 (100)	Negative	691	28	719	93.23	32.47	0.454	0.383–0.525	2.3205E‐36	Moderate agreement
				Positive	24	25	49						
				Total	715	53	768						
	Total (1600)	1531 (95.69)	1574 (98.38)	Negative	1382	39	1421	94.82	36.59	0.508	0.458–0.559	1.5124E‐86	Moderate agreement
				Positive	39	45	84						
				Total	1421	84	1505						

*Note*: *N* = 1505 women; accepted both HCP‐collected samples and self‐collected samples.

Abbreviations: CI, confidence interval; HCP, health care personnel; HEP, health education program; HPV, human papillomavirus.

Prevalence of HPV was 7.81% (95% CI = 6.545%–9.238%). The prevalence was similar across settings (7.00% (95% CI = 4.924%–9.601%) in urban non‐slum, 7.80% (95% CI = 5.605%–10.509%) in urban slum and 8.50% (95% CI = 6.394%–11.025%) in rural. In total, 125 women were positive on either test; 85 each on SS and HCP collected sample. Ninety‐six women (76.80%) complied for further clinical assessment after positive HPV test. Compliance was least among rural women (68.63%), followed by urban non‐slum women (77.14%) and was best among urban slum women (87.18%). Colposcopy was performed among 96 women who complied for referral, lesions were identified on Colposcopy among 25 (26%) women, and hence cervical biopsies were undertaken. On histopathology examination, 5 women were diagnosed with CIN 1, 1 women with CIN 2, and 2 women with CIN 3. All except one woman completed the treatment.

The test characteristics of HPV‐SS and HPV by HCP in terms of sensitivity, specificity, FPR, FNR, PPV, and NPV were 87.50%, 95.02%, 4.98%, 12.50%, 8.24%, 99.93% and 87.50%, 94.88%, 5.12%, 12.50%, 8.24%, 99.93%, respectively at cutoff of CIN1 and 66.67%, 94.72%, 5.28%, 33.33%, 2.35%, 99.93% and 66.67%, 94.57%, 5.43%, 33.33%, 2.35%, 99.93% at cutoff of ≥CIN 2, respectively.

## 4 DISCUSSION

In the current trial, the median age at enrollment of women was 40 years. This is the best age group to be targeted for cervical cancer screening. According to WHO guidelines, if a woman can be screened only once in her lifetime, then the recommended age group is between 35 and 45 years.^22^

In this study, illiteracy was maximum among rural women (13.50%) followed by women from urban slums (11.40%). According to NFHS 5 survey of Maharashtra State, 76.9% of rural women and 88.2% of urban women were literate. The median age of marriage was 22, 19, and 20 years among the urban non‐slum, urban slum, and rural women in this study. The NFHS 5 survey shows that 15.7% of urban girls and 27.6% of rural girls get married before 18 years in Maharashtra. Also, the survey shows that 65.8% of urban women and 66.5% of rural women use some family planning method. This was around 51% in this study.^23^

In this study, the knowledge regarding causes, symptoms and test for early detection of cervical cancer was poor in the pre‐intervention period. Similar findings were noted among community health workers of Varanasi district, Uttar Pradesh.^24^ An Indonesian study demonstrates that only 53.6% of women had knowledge regarding cervical cancer screening^25^ while in Brazil^26^ 79.44% women were aware of persistent HPV infection leading to cervical cancer. Several studies conducted in different regions in India have reported varying levels of knowledge about cervical cancer screening ranging from just 3.25% in North India as reported by Pattupara et al.,^27^ 32% by Siddharthar et al.^28^ in rural Puducherry, 47% among women in rural South Tamil Nadu^29^ and 84% in another Puducherry study.^30^ A Haryana study reported poor knowledge about cervical cancer, its screening and about HPV infection among majority of women in rural areas as compared to urban areas.^31^

A total of 73% women in this study thought it was necessary to get screened even in absence of symptoms, reflecting their attitude toward screening. Around 78% women in this study thought that any women could get cervical cancer. Despite this only 5% women had undergone prior cervical cancer screening. The reasons for poor prior screening uptake could be multifactorial, including, lack of deeper understanding regarding causes and symptoms, (Figure 2) difficulty in access and availability of only limited number of screening facilities, stigma, cultural barriers—including embarrassment associated with gynecological examination, patriarchal social system with inequitable gender‐based power dynamics where males are the decision makers, even regarding women accessing a health care facility and social and economic dependency on men. NFHS 5 survey records 1.9% women being ever screened for cervical cancer.^2^ In this study, screening was mainly advised by family Physician and community health workers. A cross‐sectional study among nurses in Sikkim found that awareness about Pap smear testing primarily came from physicians, followed by Medical textbooks.^32^ A survey at Bhopal Hospital showed favorable attitude toward cervical cancer screening among 76.25% women.^33^ A similar survey by Singh et al.^34^ at a Delhi hospital, reported positive attitude toward screening only among 18.2% women.

There was a significant improvement in knowledge and attitude toward cervical cancer and cervical cancer screening in the post intervention period. Similar findings were noted among paramedical professionals in Mumbai following a novel educational training for cervical cancer screening.^35^ In this study, 4% women refused examination by HCP to collect HPV samples. In Barshi district, during 1999–2003, 78% eligible women participated in cervical cancer screening.^36^

Self‐sampling was accepted by 99.2% of urban non‐slum women, 97% of urban slum women, and 98.8% of rural women. Acceptance was similar among women who received information with health education and among women who received information with pamphlets except that among urban slum women who received information with pamphlets, the acceptance was only 88.4% versus those who received information regarding self‐collection by HEP wherein acceptance was 98%. In a study by Perez et al.^26^ over 60% of participants expressed preference for a speculum‐free examination, 80% were willing to try HPV DNA self‐sampling and 63% were agreeing cervix self‐visualization. These findings highlight importance of self‐testing methods that provide women with increased accessibility, autonomy and control during the examination. More women accepted self‐sampling (1574) as compared to HCP collected samples (1531) in this study. Participation in cervical cancer screening increased from 72.6% to 82.2% in a Finnish screening program that used a reminder letter followed by mailing of self‐sampling kit.^37^ A 2017 meta‐analysis by Nelson et al.^16^ demonstrates that self‐sampling is generally well‐received. Another meta‐analysis of nine studies reported 87% women willing to undertake self‐sampling in the future.^16^ Further, a systematic review by Morgan et al.,^38^ showed preference for self‐sampling by women compared to clinician‐collected sampling (64.7%–93%).

In this study among women who refused self‐sampling, many women expressed that they would feel more confident if the test was performed by HCP. Morgan et al.^38^ found that women preferred clinician‐collected sampling because they lacked confidence in their ability to properly perform self‐sampling. Women in this study were also concerned about test being painful and hurting self. Similar concerns about hurting themselves were raised by women in a study by Bansil et al.,^39^ among women in Hyderabad and Nicaragua. While women in Uganda were most concerned about not obtaining a good sample. These two worries were commonly observed across all the surveyed sites.

Overall, moderate agreement was seen between the HPV‐ and HCP‐collected samples and HPV‐SS in all settings and irrespective of modality of communication in the present trial. Similarly, moderate agreement was noted in Thailand study^40^ and good concordance was seen in a Japanese study with self‐sampling.^41^ A meta‐analysis involving 26 studies reported a pooled overall agreement of 88.7%; with positive agreement of 84.6%, negative agreement of 91.7%, and kappa value of .72.^42^

Prevalence of HPV in this study was 7.81% and was similar across settings. An IARC study showed variation in age‐standardized HPV prevalence between populations, ranging from 1.4% in Spain to 25.6% in Nigeria.^43^ Another study from the Dindigul district of Tamil Nadu found HPV prevalence of 16.9%.^44^ The prevalence of HPV was reported to be 10.3% in a study conducted in villages of Osmanabad in 2005.^45^ In this study, 76.8% of screen‐positive women complied for referral. Dinshaw et al.^46^ reported 79% compliance to referral after a positive cervical cancer screening test in Mumbai.

Fairly good sensitivity, specificity, and NPV are observed both for self‐ samples and HCP samples at cut off of both CIN1 and ≥CIN 2 in this study. In a Japanese study, sensitivity of CIN2+ detection was 100% by both SS and Physician sampling. Specificity was 58.1% and 57%, PPV was 26.5 and 26% and NPV was 100% for SS and Physician sampling, respectively.^41^

Some of the limitations of the study are, post‐intervention information was collected mostly within a day and that might have led to short‐term retention of information, reflected as increase in knowledge. This was done in order to reduce attrition. Another limitation is that the use of combined modality (pamphlets and health education) has not been explored. The study does not reflect comprehensive understanding of effectiveness of HPV self‐sampling, as the long‐term follow‐up data of the impact of self‐sampling on cervical cancer rates and its integration into routine screening programs were not part of the trial objectives. This study does not include the Qualitative research component that could have explored the reasons behind the identified barriers and the overall experience of women using self‐sampling. Cost‐effectiveness analysis was not part of this study and hence the economic implications and potential for scalability of HPV self‐sampling in resource‐limited settings could not be explored. Finally, this study was conducted in one State in India, though in different settings. Whether these results would apply to the entire country which is so diverse, will need to be verified.

This study conducted in varied settings in India and using different modalities of communication for creating awareness and explaining the procedure of collecting self‐samples clearly demonstrates good acceptability, feasibility, validity of self‐sampling and good agreement between the self‐sampling and HCP collected samples for HPV testing.

## AUTHOR CONTRIBUTIONS

**Gauravi A. Mishra:** Conceptualization; methodology; supervision; project administration; funding acquisition; writing—original draft; writing—review and editing; investigation; resources; visualization; validation. **Sharmila A. Pimple:** Writing—review and editing; supervision. **Kavita V. Anand:** Project administration; resources; supervision; validation; visualization; writing—review and editing. **Vasundhara Y. Kulkarni:** Project administration; resources; supervision; visualization; validation; writing—review and editing. **Anil S. Patil:** Formal analysis; software; data curation; visualization. **Sanjay K. Biswas:** Writing—review and editing; investigation; validation.

## FUNDING INFORMATION

The trial was wholly supported by funds from Terry Fox Foundation (Grant Number I1050).

## CONFLICT OF INTEREST STATEMENT

We declare that we have no conflict of interest.

## ETHICS STATEMENT

This study involves human participants and was approved by the Institutional Ethics Committee (IEC) of Tata Memorial Hospital, IEC No. 1686. Informed consent was taken for all participants. The trial was registered with Clinical Trials Registry – India (CTRI) https://www.ctri.nic.in with CTRI No. – CTRI/2020/09/027748. (Find by Keywords – CTRI/2020/09/027748).

## Supporting information


**Table S1:** Detailed demographic data.
**Table S2:** Overall comparison of pre‐intervention and post‐intervention knowledge and attitudes toward cervical cancer and screening.
**Table S3:** Distribution of self‐sampling acceptance by literacy level across settings.

## Data Availability

The data that support the findings of this study are available from the corresponding author upon reasonable request.
