# Supplementary material for: Acceptability and validity of HPV self‐sampling for cervical cancer screening among women living in different ecological settings in India
Source: Int J Cancer. 2024 Oct 16;156(6):1142–53. doi: 10.1002/ijc.35222 (PMC11737002; doi:10.1002/ijc.35222)
Supplement: Supplementary file 1 — Table S1: Detailed demographic data. Table S2: Overall comparison of pre‐intervention and post‐intervention knowledge and attitudes toward cervical cancer and screening. Table S3: Distribution of self‐sampling acceptance by literacy level across settings. [file IJC-156-1142-s001.pdf]

**Title: Acceptability and validity of HPV self-sampling for cervical cancer screening among women living in different ecological settings in India.**

**Author list:**

**Gauravi A Mishra, Sharmila A Pimple, Kavita V Anand, Vasundhara Y Kulkarni, Anil S Patil, Sanjay K Biswas**

**Table of Contents of Supplementary Material:**


| Sr. No. | Supplementary Material | Title |
|---|---|---|
| 1. | Supplementary Table 1 | Detailed Demographic Data |
| 2. | Supplementary Table 2 | Overall comparison of pre-intervention and post-intervention knowledge and attitudes towards cervical cancer and screening |
| 3. | Supplementary Table 3 | Distribution of Self-Sampling Acceptance by Literacy Level Across Settings |

## Supplementary Tables:

### Supplementary Table 1: Detailed Demographic Data

| Variability | Overall N (%) 1600 | | P – Value# | Urban Non-Slum n (%) 500 | | P – Value# | Urban Slum n (%) 500 | | P – Value# | Rural n (%) 600 | | P – Value# |
|---|---|---|---|---|---|---|---|---|---|---|---|---|
| | HEP Arm | P Arm | | HEP Arm | P Arm | | HEP Arm | P Arm | | HEP Arm | P Arm | |
| **Age** | | | | | | | | | | | | |
| 30-35 | 263 (32.88) | 219 (27.38) | 0.1927 | 62 (24.80) | 57 (22.80) | 0.5518 | 107 (42.80) | 74 (29.60) | 0.0177 | 94 (31.33) | 88 (29.33) | 0.7305 |
| 36-40 | 203 (25.37) | 211 (26.38) | | 70 (28.00) | 58 (23.20) | | 57 (22.80) | 80 (32.00) | | 76 (25.33) | 73 (24.33) | |
| 41-45 | 153 (19.13) | 166 (20.75) | | 57 (22.80) | 60 (24.00) | | 37 (14.80) | 50 (20.00) | | 59 (19.67) | 56 (18.67) | |
| 46-50 | 117 (14.63) | 133 (16.63) | | 38 (15.20) | 44 (17.60) | | 35 (14.00) | 32 (12.80) | | 44 (14.67) | 57 (19.00) | |
| 51-55 | 64 (8.00) | 71 (8.88) | | 23 (9.20) | 31 (12.40) | | 14 (5.60) | 14 (5.60) | | 27 (9.00) | 26 (8.67) | |
| Mean age (SD) | 39.74 (6.83) | 40.53 (6.69) | 0.0195$ | 40.77 (6.61) | 41.61 (6.91) | 0.1656$ | 38.48 (6.76) | 39.60 (6.22) | 0.0528$ | 39.95 (6.91) | 40.41 (6.77) | 0.4036$ |
| Median age (IQR) | 39 (34 - 45) | 40 (35 - 46) | | 40 (36 - 45) | 42 (36 - 47) | | 37 (33 - 44) | 39 (35 - 44) | | 39 (35 - 45) | 40 (35 - 46) | |
| Mean age at marriage (SD) | 20.86 (4.40) | 20.98 (4.55) | 0.5916$ | 22.99 (4.64) | 22.92 (4.88) | 0.8585$ | 19.60 (4.06) | 20.28 (4.19) | 0.0690$ | 20.12 (3.78) | 19.95 (4.02) | 0.5796$ |

| | | | | | | | | | | | | |
|---|---|---|---|---|---|---|---|---|---|---|---|---|
| Median age at marriage (IQR) | 20 (18 - 23) | 20 (18 - 24) | | 22 (20 - 26) | 22.5 (19 - 25) | | 19 (17 - 22) | 19.5 (18 - 22) | | 20 (18 - 22) | 19 (17 - 23) | |
| **Education** | | | | | | | | | | | | |
| Illiterate/ Literate without formal education | 68 (8.50) | 83 (10.38) | 0.4141 | 7 (2.80) | 6 (2.40) | 0.8705 | 25 (10.00) | 32 (12.80) | 0.2458 | 36 (12.00) | 45 (15.00) | 0.5966 |
| Primary (1-4) | 51 (6.38) | 43 (5.38) | | 8 (3.20) | 9 (3.60) | | 22 (8.80) | 16 (6.40) | | 21 (7.00) | 18 (6.00) | |
| Secondary (5-10) | 475 (59.38) | 495 (61.88) | | 127 (50.80) | 133 (53.20) | | 147 (58.80) | 158 (63.20) | | 201 (67.00) | 204 (68.00) | |
| Higher secondary (11-12) | 103 (12.88) | 83 (10.38) | | 44 (17.60) | 38 (15.20) | | 27 (10.80) | 22 (8.80) | | 32 (10.67) | 23 (7.67) | |
| Sr. College (13-15) undergraduates | 48 (6.00) | 46 (5.75) | | 27 (10.80) | 33 (13.20) | | 19 (7.60) | 9 (3.60) | | 2 (0.67) | 4 (1.33) | |
| Graduates and above | 55 (6.88) | 50 (6.25) | | 37 (14.80) | 31 (12.40) | | 10 (4.00) | 13 (5.20) | | 8 (2.67) | 6 (2.00) | |
| **Religion by birth** | | | | | | | | | | | | |
| Hindu | 688 (86.00) | 684 (85.50) | 0.6593 | 247 (98.80) | 248 (99.20) | 0.8456 | 157 (62.80) | 147 (58.80) | 0.1022 | 284 (94.67) | 289 (96.33) | 4057 |
| Muslim | 18 (2.25) | 14 (1.75) | | 0 (0.00) | 0 (0.00) | | 10 (4.00) | 6 (2.40) | | 8 (2.67) | 8 (2.67) | |
| Buddhist | 88 (11.00) | 92 (11.50) | | 1 (0.40) | 1 (0.40) | | 82 (32.80) | 90 (36.00) | | 5 (1.67) | 1 (0.33) | |

| | | | | | | | | | | | | |
|---|---|---|---|---|---|---|---|---|---|---|---|---|
| Christian/Jain/Sikh | 6 (0.75) | 10 (1.25) | | 2 (0.80) | 1 (0.40) | | 1 (0.40) | 7 (2.80) | | 3 (1.00) | 2 (0.67) | |
| **Occupation** | | | | | | | | | | | | |
| House wife | 624 (78.00) | 591 (73.88) | 0.2927 | 188 (75.20) | 187 (74.80) | 0.0229 | 198 (79.20) | 193 (77.20) | 0.8194 | 238 (79.33) | 211 (70.33) | 0.0348 |
| Manual Labour | 90 (11.25) | 107 (13.38) | | 25 (10.00) | 10 (4.00) | | 22 (8.80) | 27 (10.80) | | 43 (14.33) | 70 (23.33) | |
| Service | 70 (8.75) | 83 (10.38) | | 29 (11.60) | 39 (15.60) | | 28 (11.20) | 29 (11.60) | | 13 (4.33) | 15 (5.00) | |
| Self employed | 16 (2.00) | 19 (2.38) | | 8 (3.20) | 14 (5.60) | | 2 (0.80) | 1 (0.40) | | 6 (2.00) | 4 (1.33) | |
| **Monthly Family income (in Rs.)** | | | | | | | | | | | | |
| <=10,000 | 404 (50.50) | 395 (49.38) | 0.3446## | 98 (39.20) | 91 (36.40) | 0.2900## | 111 (44.40) | 86 (34.40) | 0.0140## | 195 (65.00) | 218 (72.67) | 0.0262## |
| >10,000 | 396 (49.50) | 405 (50.63) | | 152 (60.80) | 159 (63.60) | | 139 (55.60) | 164 (65.60) | | 105 (35.00) | 82 (27.33) | |

**#:** Pearson's Chi-squared test; ##: Fisher's Exact Test, $:Independent Samples t-Test

***HEP: Health Education Programme;**P: Pamphlet**

**Supplementary Table 2: Overall comparison of pre-intervention and post-intervention knowledge and attitudes towards cervical cancer and screening**

| Category | Variable | Pre Intervention | Post Intervention | P-value# |
|---|---|---|---|---|
| Knowledge | Causes of cervical cancer_infection with virus | 40 (2.50) | 1442 (90.13) | 1.18E-72 |
| | Causes of cervical cancer_women with many children | 612 (38.25) | 1573 (98.31) | 5.70E-306 |
| | Causes of cervical cancer_women using tobacco | 1247 (77.94) | 1573 (98.31) | 1.47E-210 |
| | Symptoms of cervical cancer_intermenstrual bleeding | 904 (56.50) | 1518 (94.88) | 1.95E-72 |
| | Symptoms of cervical cancer_post menopausal bleeding | 306 (19.13) | 994 (62.13) | 1.11E-134 |
| | Symptoms of cervical cancer_post coital bleeding | 133 (8.31) | 786 (49.13) | 8.13E-148 |
| | Test available for early detection cervical cancer | 117 (7.31) | 1589 (99.31) | 9.85E-143 |
| | HPV virus | 19 (1.19) | 1517 (94.81) | <0.0001 |
| Attitude | Think that any woman can get cervical cancer | 1254 (78.38) | 1581 (98.81) | <0.0001 |
| | Think cervical cancer screening is important? | 1298 (81.13) | 1598 (99.88) | 8.97E-67 |
| | Feel you should carry out the test for cervical cancer detection in absence of any symptoms? | 1168 (73.00) | 1565 (97.81) | 6.74E-88 |
| | Willing to participate in cervical cancer screening if made available free of cost | 1600 (100.00) | 1600 (100.00) | 1.0000 |
| | Comfortable in collecting your own vaginal sample at home, if we teach you how to do it | 1568 (98.00) | 1570 (98.13) | 0.8802 |

#: Mc-Nemar test

**Supplementary Table 3: Distribution of Self-Sampling Acceptance by Literacy Level Across Settings**

| | **Overall N (%) 1600** | | | **P – Value#** | **Urban Non-Slum n (%) 500** | | | **P – Value#** | **Urban Slum n (%) 500** | | | **P – Value#** | **Rural n (%) 600** | | | **P – Value#** |
|---|---|---|---|---|---|---|---|---|---|---|---|---|---|---|---|---|
| | Acceptance n=1574 | Rejection N=26 | Total N=1600 | | Acceptance N=496 | Rejection N=4 | Total N=500 | | Acceptance N=485 | Rejection n=15 | Total N=500 | | Acceptance N=593 | Rejection n=7 | Total N=600 | |
| Education | | | | | | | | | | | | | | | | |
| Illiterate/ Literate without formal education | 145 (9.21) | 6 (23.08) | 151 (9.44) | 0.1124 | 13 (2.62) | 0 (0.00) | 13 (2.60) | 0.8682 | 55 (11.34) | 2 (13.33) | 57 (11.40) | 0.5385 | 77 (12.98) | 4 (57.14) | 81 (13.50) | 0.0222 |
| Primary (1-4) | 92 (5.84) | 2 (7.69) | 94 (5.88) | | 17 (3.43) | 0 (0.00) | 17 (3.40) | | 37 (7.63) | 1 (6.67) | 38 (7.60) | | 38 (6.41) | 1 (14.29) | 39 (6.50) | |
| Secondary (5-10) | 954 (60.61) | 16 (61.54) | 970 (60.62) | | 258 (52.02) | 2 (50.00) | 260 (52.00) | | 293 (60.41) | 12 (80.00) | 305 (61.00) | | 403 (67.96) | 2 (28.57) | 405 (67.50) | |
| Higher secondary (11-12) | 186 (11.82) | 0 (0.00) | 186 (11.63) | | 82 (16.53) | 0 (0.00) | 82 (16.40) | | 49 (10.10) | 0 (0.00) | 49 (9.80) | | 55 (9.27) | 0 (0.00) | 55 (9.17) | |
| Sr College (13-15) undergraduates | 93 (5.91) | 1 (3.85) | 94 (5.88) | | 59 (11.90) | 1 (25.00) | 60 (12.00) | | 28 (5.77) | 0 (0.00) | 28 (5.60) | | 6 (1.01) | 0 (0.00) | 6 (1.00) | |
| Graduates and above | 104 (6.61) | 1 (3.85) | 105 (6.56) | | 67 (13.51) | 1 (25.00) | 68 (13.60) | | 23 (4.74) | 0 (0.00) | 23 (4.60) | | 14 (2.36) | 0 (0.00) | 14 (2.33) | |
| **Religion** | | | | | | | | | | | | | | | | |
| Hindu | 1355 (86.09) | 17 (65.38) | 1372 (85.75) | 0.0019 | 491 (98.99) | 4 (100.00) | 495 (99.00) | 0.9798 | 298 (61.44) | 6 (40.00) | 304 (60.80) | 0.1879 | 566 (95.45) | 7 (100.00) | 573 (95.50) | 0.9536 |

| | | | | | | | | | | | | | | | | |
|---|---|---|---|---|---|---|---|---|---|---|---|---|---|---|---|---|
| Muslim | 32 (2.03) | 0 (0.00) | 32 (2.00) | | 0 (0.00) | 0 (0.00) | 0 (0.00) | | 16 (3.30) | 0 (0.00) | 16 (3.20) | | 16 (2.70) | 0 (0.00) | 16 (2.67) | |
| Buddhist | 171 (10.86) | 9 (34.62) | 180 (11.25) | | 2 (0.40) | 0 (0.00) | 2 (0.40) | | 163 (33.61) | 9 (60.00) | 172 (34.40) | | 6 (1.01) | 0 (0.00) | 6 (1.00) | |
| Christian/Jain/Sikh | 16 (1.02) | 0 (0.00) | 16 (1.00) | | 3 (0.60) | 0 (0.00) | 3 (0.60) | | 8 (1.65) | 0 (0.00) | 8 (1.60) | | 5 (0.84) | 0 (0.00) | 5 (0.83) | |
| **Occupation** | | | | | | | | | | | | | | | | |
| House wife | 1191 (75.67) | 24 (92.31) | 1215 (75.94) | 0.1822 | 372 (75.00) | 3 (75.00) | 375 (75.00) | 0.8387 | 377 (77.73) | 14 (93.33) | 391 (78.20) | 0.5002 | 442 (74.54) | 7 (100.00) | 449 (74.83) | 0.4970 |
| Manual Labour | 197 (12.52) | 0 (0.00) | 197 (12.31) | | 35 (7.06) | 0 (0.00) | 35 (7.00) | | 49 (10.10) | 0 (0.00) | 49 (9.80) | | 113 (19.06) | 0 (0.00) | 113 (18.83) | |
| Service | 151 (9.59) | 2 (7.69) | 153 (9.56) | | 67 (13.51) | 1 (25.00) | 68 (13.60) | | 56 (11.55) | 1 (6.67) | 57 (11.40) | | 28 (4.72) | 0 (0.00) | 28 (4.67) | |
| Self employed | 35 (2.22) | 0 (0.00) | 35 (2.19) | | 22 (4.44) | 0 (0.00) | 22 (4.40) | | 3 (0.62) | 0 (0.00) | 3 (0.60) | | 10 (1.69) | 0 (0.00) | 10 (1.67) | |
| **Income** | | | | | | | | | | | | | | | | |
| <=10,000 | 786 (49.94) | 13 (50.00) | 799 (49.94) | 0.5756## | 188 (37.90) | 1 (25.00) | 189 (37.80) | 0.5133## | 190 (39.18) | 7 (46.67) | 197 (39.40) | 0.3702## | 408 (68.80) | 5 (71.43) | 413 (68.83) | 0.6207## |
| >10,000 | 788 (50.06) | 13 (50.00) | 801 (50.06) | | 308 (62.10) | 3 (75.00) | 311 (62.20) | | 295 (60.82) | 8 (53.33) | 303 (60.60) | | 185 (31.20) | 2 (28.57) | 187 (31.17) | |

**#:** Pearson's Chi-squared test; ##: Fisher’s Exact Test,
